# A new family with a homozygous nonsense variant in *NTHL1* further delineated the clinical phenotype of *NTHL1*-associated polyposis

**DOI:** 10.1038/s41439-019-0077-3

**Published:** 2019-10-10

**Authors:** Mays Altaraihi, Anne-Marie Gerdes, Karin Wadt

**Affiliations:** Department of Clinical Genetics, Copenhagen University Hospital Rigshospitalet, Copenhagen, Denmark

**Keywords:** Colon cancer, Cancer genetics

## Abstract

A new family with *NTHL1*-associated polyposis (NAP) is described, involving a 58-year-old male affected with >100 colorectal polyps and a 55-year-old female sibling with nine colorectal polyps. The female was also diagnosed with a thyroid adenoma at age 40. Significantly, no malignant neoplasms have been detected in this family, which is important to further delineate the clinical phenotype related to NAP. A review of previously reported obligate heterozygous carriers of *NTHL1* variants showed two patients affected with neoplasms at <55 years of age, generating a study to outline the phenotypic spectrum in patients with heterozygous pathogenic *NTHL1* variants relevant.

Colorectal adenomatous polyposis syndromes can be divided into familial adenomatous polyposis (FAP), *MUTYH*-associated polyposis (MAP), polymerase proofreading-associated polyposis (PPAP) and *NTHL1*-associated polyposis (NAP).

FAP is responsible for approximately 1% of all CRCs^1^ and is due to pathogenic variants in the *adenomatous polyposis coli* (*APC)* gene. The *APC* gene is the most frequently mutated gene associated with colorectal adenomatous polyposis. The *MUTYH* gene, which is involved in the base excision repair (BER) pathway, is the second most commonly mutated gene associated with adenomatous polyposis. In contrast to FAP and PPAP, MAP shows recessive inheritance.

Recently, Weren et al. uncovered another gene in the BER pathway, *NTHL1*, associated with adenomatous polyposis and recessive inheritance^2^. Apart from adenomatous polyposis, the occurrence of ovarian cancer, duodenal cancer, basal cell carcinoma, breast cancer, endometrial malignancy, meningioma, and bladder cancer have been reported in individuals with loss-of-function variants in *NTHL1*^3^.

We report a new family with NAP:

## Patient 1

**Fig. 1:**
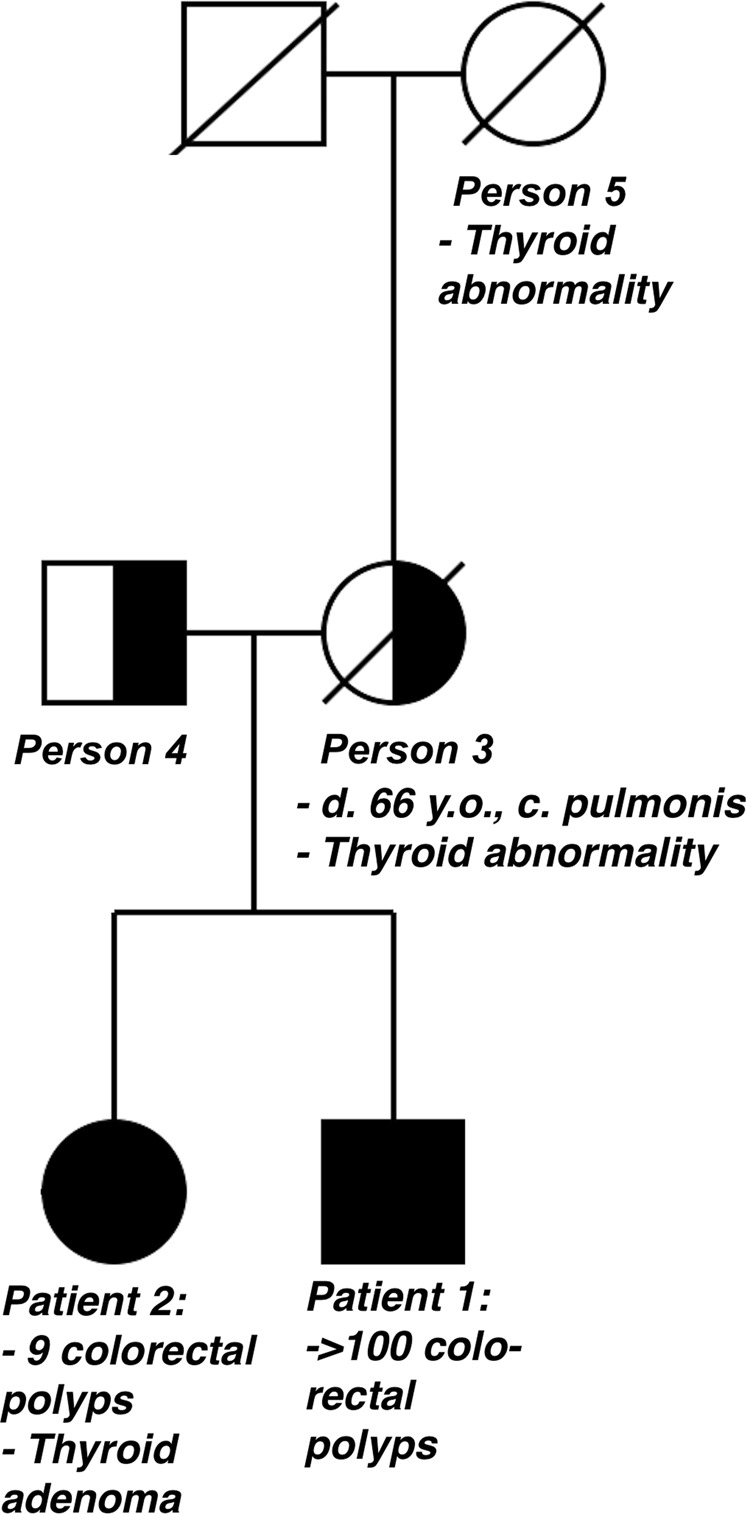
The pedigree shows the first identified Danish family carrying a homozygous nonsense variant in *NTHL1*

A 47-year-old man (patient 1) developed hematochezia triggered by a gastrointestinal infection (Fig. 1). A colonoscopy was performed, identifying 23 polyps; three in the caecum, the rest in the left colon. Polypectomy was performed, and a pathology review of eight polyps showed six tubular adenomas with either low or moderate dysplasia and two tubulovillous adenomas, one of which had severe dysplasia. The largest adenoma measured 20 mm × 8 mm × 15 mm.

The patient had no family history of colonic adenomas or cancer, and genetic testing was not performed. However, the patient was enrolled for routine screening, and during a 10-year span (2007–2017), annual colonoscopic polypectomies identified and removed approximately 40 polyps. Approximately eighty percent of the polyps were adenomas.

In June 2018, at age 58, a colonoscopy was performed, identifying and removing 55 new polyps that had developed during the preceding 6 months. Four of the five pathologically described polyps were adenomas; the last polyp was identified as a sessile serrated polyp.

The most recent colonoscopy, performed in December 2018, showed 20 polyps, in which all pathologically described polyps were tubular adenomas with low dysplasia. The patient has developed 133 polyps so far and has declined undergoing a recommended colectomy. He was referred to genetic counseling, and an analysis of a colorectal cancer gene panel consisting of 17 genes was performed (*APC, BMPR1A, CDH1, EPCAM, GREM1, MLH1, MSH2, MSH6, MUTYH, NTHL1, PMS2, POLD1, POLE, PTEN, SMAD4, STK11, TP53*). A previously reported homozygous nonsense variant (NM_002528):c.268C>T, p. Gln90Ter in the *NTHL1* gene was identified. No alterations were found in the other examined genes.

As other cancer types have been described in patients with homozygous nonsense variants in *NTHL1*, a gastroscopy and a dermatological examination were performed, finding no suspicious results. A urine cytology test showed no malignant cells.

## Patient 2

The 55-year-old sister of patient 1 presented with a thyroid adenoma at age 40, which was later characterized as a cold nodule.

Due to multiple colorectal polyps in her brother, the patient underwent a colonoscopy, and a tubular adenoma with moderate dysplasia was removed from the caecum at age 45. She was referred to genetic counseling, and genetic testing identified the same homozygous nonsense variant, c.268C>T, in *NTHL1*.

A gastroscopy and a dermatological examination revealed no suspicious findings.

A recent colonoscopy showed eight colorectal polyps, which were removed. Four polyps were pathologically reviewed, classifying three as tubular adenomas with low dysplasia and one sessile serrated polyp. Mammography and gynecological examination were conducted, revealing no signs of breast or endometrial cancer, respectively.

## Other family members

Person 3, the mother of patients 1 and 2, is an obligate heterozygous carrier of the nonsense variant, c.268 C>T, in *NTHL1*. At age 58, she was diagnosed with pulmonary squamous cell carcinoma, having a smoking history of 67.5 pack-years. She had also undergone thyroidectomy due to goiter, approximately at age 50. The patient died of lung cancer at 66 years old.

Person 4, the father of patients 1 and 2, is also an obligate heterozygous carrier of the *NTHL1* variant and has no neoplasms or thyroid-associated diseases.

The offspring of patients 1 and 2 are obligate heterozygous carriers and are all healthy.

This study reports the first family with a homozygous nonsense variant in the *NTHL1* gene in Denmark. Globally, 18 families (30 individuals) with biallelic *NTHL1* variants have been reported^4^. The majority (*N* = 23) of the reported patients are homozygous for the same nonsense variant: c.268C>T. Five of the reported patients are compound heterozygous individuals. Four of them have the c.268C>T, *NTHL1* variant (Table 1 in ref. ^5^). c.268C>T is the most frequent loss-of-function variant in *NTHL1*, with an allele count of 400 (allele frequency of 0.14%) according to gnomAD browser (*N* = 141,111 exomes), all in a heterozygous state, with the highest frequency in the European Finnish population (0.35%) (http://gnomad.broadinstitute.org/variant/16-2096239-G-A).

This family further expands the phenotypic spectrum linked to *NTHL1*-associated polyposis and raises the total number of families to 19 (32 individuals).

Previously, one individual with biallelic variants in *NTHL1* has been reported with thyroid cancer, diagnosed at age 60^5^. Patient 2 in this study, a homozygous carrier of the pathogenic *NTHL1* variant, developed thyroid adenoma at age 40. Her mother (person 3) had undergone a thyroidectomy due to a thyroid abnormality. No specification of the thyroid abnormality can be made as no medical records or tissue from the thyroidectomy of person 3 can be found.

Papillary thyroid cancer, thyroid nodules, and multinodular goiter have been reported in patients with FAP and MAP^6,7^, suggesting a link between thyroid abnormalities and NAP, although disorders of the thyroid gland can be associated with several other genetic diseases, which has not been analyzed in this family.

Moreover, studies have shown that heterozygous carriers of pathogenic *MUTYH* variants have twice the population risk of developing colorectal adenomas and endometrial cancer and three times the population risk of developing liver and gastric cancer^8^. One study suggested no increased risk of developing colorectal adenomas in individuals heterozygous for the pathogenic *NTHL1* variant^9^. None of the heterozygous carriers in the current family had a colonoscopy performed.

However, reported manifestations found in obligate *NTHL1* heterozygous carriers in the literature may suggest a heightened risk of developing other neoplasms. Significantly, one female was diagnosed with an endometrial tumor at age 41, and one male was diagnosed with gastric cancer at age 53. One heterozygous female carrier of the c.268C>T variant was diagnosed with two undescribed tumors. The number of *NTHL1* families is very limited, and only a few *NTHL1* heterozygous patients are reported with symptoms, so no definite conclusion can be drawn.

Patient 1 developed 133 colorectal polyps by the age of 58, 55 of which developed during a 6-month period. This observation is important when considering the natural history of colorectal polyposis associated with *NTHL1* mutations and corroborates the current recommendation of colectomy when a considerable number of adenomas are identified. This case report is the second to describe a patient with a pathogenic homozygous *NTHL1* variant affected with >100 colorectal polyps, both being males^10^. Previously, only four reported biallelic *NTHL1* variant carriers have been described without malignant neoplasms, raising the number to six out of 32 patients without any malignant neoplasms when including the siblings. This observation is certainly age-dependent, and both reported patients have also been under clinical surveillance with short intervals, which may contribute to finding no malignant neoplasms.

In conclusion, none of the reported siblings, aged 55 and 58 years, developed malignant neoplasms; however, the median age at diagnosis for colorectal cancer in *NTHL1* homozygous or compound heterozygous carriers is 59 years for men and 64 years for women^5^.

Moreover, we have observed thyroid abnormalities in both heterozygote and homozygote carriers in this family. Studies of larger cohorts of individuals with *NTHL1* variants in both heterozygous and homozygous states can further delineate the phenotypic spectrum.

## Data Availability

The relevant data from this Data Report are hosted at the Human Genome Variation Database at 10.6084/m9.figshare.hgv.2606
